# Development of a monoclonal anti-ADAMTS-5 antibody that specifically blocks the interaction with LRP1

**DOI:** 10.1080/19420862.2017.1304341

**Published:** 2017-03-17

**Authors:** Salvatore Santamaria, Oleg Fedorov, John McCafferty, Gillian Murphy, Jayesh Dudhia, Hideaki Nagase, Kazuhiro Yamamoto

**Affiliations:** a Kennedy Institute of Rheumatology, Nuffield Department of Orthopaedics, Rheumatology and Musculoskeletal Sciences, University of Oxford, Headington, Oxford, UK; b Structural Genomics Consortium and Target Discovery Institute, Nuffield Department of Clinical Medicine, University of Oxford, Headington, Oxford, UK; c IONTAS, Iconix Park, Pampisford, Cambridge, UK; d Cancer Research UK Cambridge Institute, Department of Oncology, University of Cambridge, Li Ka Shing Centre, Cambridge, UK; e Department of Clinical Sciences and Services, Royal Veterinary College, North Mymms, Hatfield, Herts, UK

**Keywords:** Aggrecanase, cartilage, endocytosis, extracellular matrix, proteoglycanase

## Abstract

The potent aggrecanase ADAMTS-5 is constitutively secreted by chondrocytes, but it is rapidly endocytosed in normal cartilage *via* the cell surface endocytic receptor LRP1. Therefore it is difficult to detect the total ADAMTS-5 activity produced. In this study, we isolated a monoclonal anti-ADAMTS-5 antibody 1B7 that blocks LRP1-mediated internalization without affecting the aggrecanolytic activity. Addition of 1B7 to cultured human chondrocytes revealed the full aggrecanolytic activity of ADAMTS-5 generated by the cells. 1B7 is a useful tool to estimate the ADAMTS-5 activity and to identify its potential roles in the tissues.

## Abbreviations

ADAMTS: adamalysin-like metalloproteinases with thrombospondin motifs
AP: alkaline phosphatase
Cat: catalytic domain
CysR: Cys-rich domain
Dis: disintegrin domain
ECM: extracellular matrix
LRP1: low-density lipoprotein receptor-related protein 1
OA: osteoarthritis
RAP: receptor-associated protein
scFv: single chain variable fragment
Sp: spacer
TIMP-3: tissue inhibitor of metalloproteinase-3
TS: thrombospondin-like

## Introduction

ADAMTS-5 (a disintegrin and metalloproteinase with thrombospondin motifs 5) is a potent aggrecanase in cartilage and participates in the progression of osteoarthritis (OA).^1-5^ It is also expressed in many other tissues^6^ and cleaves extracellular matrix (ECM) proteoglycans such as versican, brevican, decorin, biglycan and fibromodulin.^7,8^ Its ability to cleave versican is considered to be important in cardiac valve development,^9^ limb morphogenesis,^10,11^ wound healing^12^ and lymphocyte trafficking following influenza virus infection.^13^

The expression of ADAMTS-5 has been reported to be regulated at the transcriptional and post-transcriptional levels,^14,15^ but we have recently found that ADAMTS-5 is constitutively produced in normal cartilage. However, it is rapidly cleared by the chondrocytes *via* the endocytic receptor, low-density lipoprotein receptor-related protein 1 (LRP1), and degraded subsequently.^16,17^ Because of its clearance, it is difficult to detect ADAMTS-5 in normal steady-state of the tissue, but it probably functions for a short, finite period of time to maintain normal turnover of cartilage matrix components.

LRP1 is a type 1 transmembrane protein consisting of a 515-kDa α-chain containing extracellular ligand-binding domains and an 85-kDa β-chain containing a transmembrane domain and a cytoplasmic domain. Both chains are derived from the same precursor and non-covalently attached on the cell surface. More than 50 ligands for LRP1 have been reported, including lipoproteins, ECM proteins, growth factors, cell surface receptors, proteinases, proteinase inhibitors and secreted intracellular proteins.^18^ Because LRP1 is widely expressed in different tissues and cell types, it may play an important role in regulating ADAMTS-5 activity not only in cartilage but also in other tissues such as blood vessels, lung, adipose tissue and brain.^6,18,19^ However, it is difficult to investigate the biologic significance of ADAMTS-5 endocytosis because there are no tools available to specifically block the endocytosis of the enzyme.

ADAMTS-5 is a multidomain metalloproteinase consisting of a catalytic (Cat), a disintegrin (Dis), a first thrombospondin-like (TS) domain, a spacer (SP) and a second C-terminal TS domain. We previously reported that the first TS and Sp domains are responsible for ADAMTS-5 binding to LRP1.^20^ The aims of this study were to isolate a monoclonal anti-ADAMTS-5 antibody that selectively blocks the interaction with LRP1 and to detect the aggrecanolytic activity of ADAMTS-5 that is masked by the endocytic clearance. We screened the battery of monoclonal antibodies for ADAMTS-5 that were isolated from a phage-displayed single-chain antibody library^17^ and obtained one that blocks ADAMTS-5 endocytosis without affecting the enzyme's aggrecanolytic activity.

## Results

**Figure 1.**
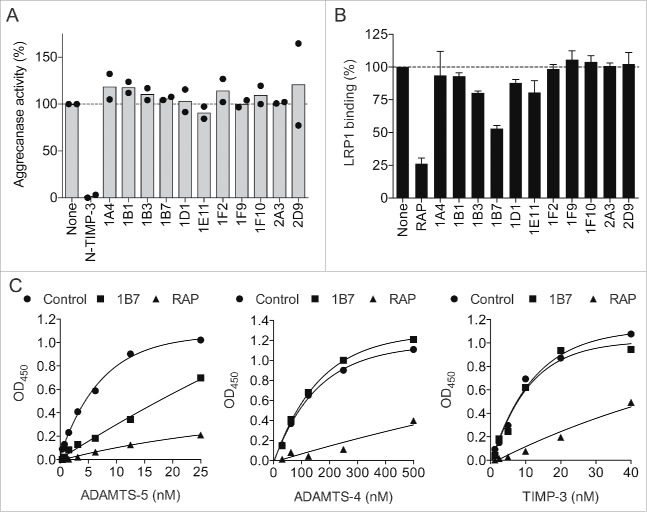
Screening of an anti-ADAMTS-5 antibody that blocks the interaction with LRP1. (A) Effect of anti-ADAMTS-5 antibodies on purified aggrecan degradation by ADAMTS-5. Purified bovine aggrecan (50 μg) was incubated with FLAG-tagged ADAMTS-5–2 (2 pM) in the absence (none) or presence of antibodies (each 500 nM) or N- TIMP-3 (10 nM) at 37°C for 2 h. The reactions were terminated with 50 mM EDTA and the reaction products were deglycosylated and subjected to Western blot analysis using anti-AGEG aggrecan neoepitope antibody. Densitometric analysis of immunoreactive bands of aggrecan fragments detected was then performed and the mean for the fragments generated in the absence of the antibodies was taken as 100%. Each point represents an individual experiment. (B) Effect of anti-ADAMTS-5 antibodies on ADAMTS-5 binding to LRP1. Full-length LRP1 was coated onto microtiter plates, and the binding of biotinylated ADAMTS-5–2 (6 nM) in the absence or presence of anti-ADAMTS-5 antibodies (each 100 nM) or RAP (100 nM) was measured using AP-conjugated streptavidin described in Section 2.4. The amount of ADAMTS-5 bound to LRP1 was expressed as % of the amount of ADAMTS-5 bound to LRP1 in the absence of antibodies or RAP. Bars represent the mean ± SD (n = 3) (C) Full-length LRP1 was coated onto microtiter plates and binding of ADAMTS-5–2 (left panel), ADAMTS-4 lacking C-terminus spacer domain (middle panel), or TIMP-3 (right panel) in the absence or presence of 1B7 or RAP (each 100 nM) was measured using anti-FLAG M2 antibody and a horseradish peroxidase-conjugate secondary antibody.

We first selected anti-ADAMTS-5 scFv-Fc antibodies (i.e. single-chain variable fragments fused with the crystallizable fragment of immunoglobulin) that have no major inhibitory effect on the aggrecanolytic activity of ADAMTS-5 from the pool of monoclonal antibodies that were previously isolated from the phage-displayed single chain antibody library.^17^ As shown in Fig. 1A, 11 anti-ADAMTS-5 antibodies did not show a major effect on the aggrecanolytic activity. We then examined them for their ability to block the binding of ADAMTS-5 to immobilized LRP1. Among them, one antibody, designated 1B7, effectively inhibited the binding of ADAMTS-5 to LRP1 and ∼50% inhibition was detected at the concentration of 100 nM (Fig. 1B). In this assay, the LRP ligand-binding antagonist, receptor-associated protein (RAP) exhibited ∼75% inhibition at 100 nM (Fig. 1B). The dose-dependent binding of ADAMTS-5 to LRP1 was markedly reduced in the presence of 100 nM 1B7, whereas the binding of ADAMTS-4 and of tissue inhibitor of metalloproteinases 3 (TIMP-3) to LRP1 was not blocked (Fig. 1C). As expected, RAP (100 nM) blocked the binding of all 3 ligands.

**Figure 2.**
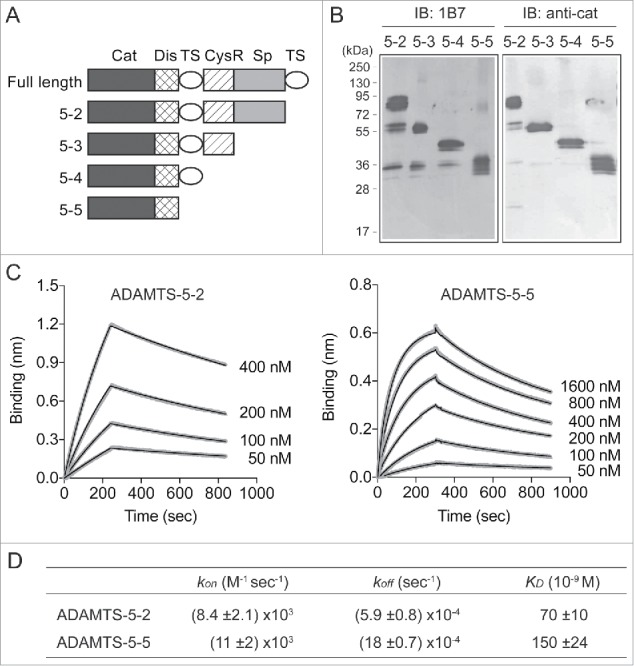
Mapping of 1B7 binding sites in the catalytic/disintegrin domains of ADAMTS-5. (A) Schematic representation of FLAG-tagged domain deletion mutants of ADAMTS-5. (B) ADAMTS-5 deletion forms (100 ng) were run on a 10% polyacrylamide gel under non-reducing conditions. 1B7 (1 µM) was used as a primary antibody and anti-human immunoglobulin AP-conjugated as a secondary antibody. For comparison, a polyclonal anti-ADAMTS-5 Cat domain antibody^8^ was used. IB: immunoblot. (C) Biolayer interferometry analysis showing the interaction of 1B7 with ADAMTS-5–2 (*left panel*) and ADAMTS-5–5 (*right panel*). Biotinylated 1B7 (1 µM) was immobilized on the surface of a streptavidin-coated biosensor and incubated with different concentrations of FLAG-tagged ADAMTS-5–2 (0–400 nM) or ADAMTS-5–5 (50–1600 nM) and the binding was analyzed in singlicate by biolayer interferometry as reported in the Method section. Fitting curves are overlaid with raw data. (D) The kinetic rate constants and *K_D_* values for binding of ADAMTS-5–2 and ADAMTS-5–5 to 1B7.

To map the 1B7 binding site in ADAMTS-5, we tested a series of domain deletion mutants of ADAMTS-5 (Fig. 2A) for their ability to bind to 1B7 by using Western blotting. As shown in Fig. 2B, all forms of ADAMTS-5 reacted with 1B7, suggesting that the antibody reacts with the Cat/Dis domains. The antibody reacted with all these forms only when the enzyme proteins were non-reduced (data not shown). Biolayer interferometry analysis showed that ADAMTS-5–2 that lacks the C-terminal TS domain bound to 1B7 with a *K*_D_ value of 70 ± 10 nM [*k*_on_: (8.4 ± 2.1) × 10^3^ M^−1^sec^−1^; *k*_off_: (5.9 ± 0.8) × 10^−4^sec^−1^] and that ADAMTS-5–5 consisting of only Cat and Dis domains (ADAMTS-5–5) bound with a *K*_D_ value of 150 ± 24 nM [*k*_on_: (11 ± 2) x 10^3^ M^−1^sec^−1^; *k*_off_: (18 ± 0.7) × 10^−4^ sec^−1^] (Fig. 2C and D).

**Figure 3.**
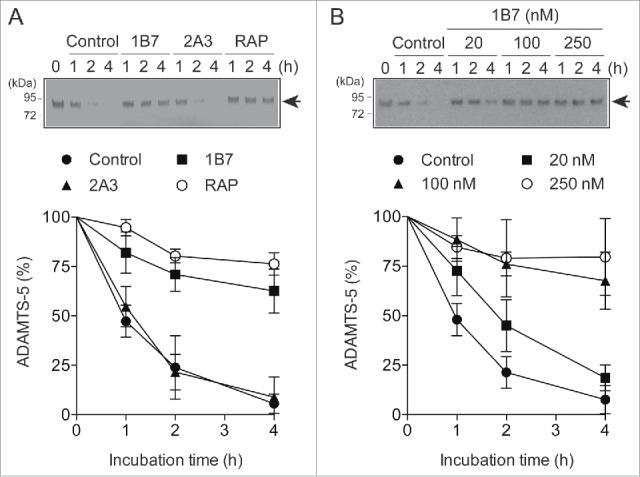
Inhibition of ADAMTS-5 endocytosis by 1B7. (A) Human chondrocytes (n = 3) were cultured with DMEM containing 10 nM of FLAG-tagged ADAMTS-5–2 in the presence or absence of 1B7 or 2A3 (each 100 nM), or RAP (500 nM) for 0–4 h. ADAMTS-5–2 in the medium was detected by Western blotting using anti-FLAG M2 antibody. *Upper panel*, representative Western blot analysis. *Lower panel*, quantified data, where the amount of ADAMTS-5 was expressed as % of the amount of the enzyme at 0 h. Points represent the mean ± SD. Arrows in the blot indicate exogenously added ADAMTS-5–2. (B) Human chondrocytes (n = 3) were cultured with DMEM containing 10 nM FLAG-tagged ADAMTS-5–2 in the presence of 1B7 (0–250 nM) for 0–4 h, and ADAMTS-5–2 in the media was detected as in (A).

To investigate whether 1B7 inhibits ADAMTS-5 endocytosis, purified ADAMTS-5 was added to the cultured human chondrocytes and the amounts of ADAMTS-5 that remained in the medium were monitored by Western blotting as described previously.^16^ As a control we used the antibody 2A3, which neither blocks the binding of ADAMTS-5 to LRP1 nor inhibits the enzyme activity (Fig. 1A and B). ADAMTS-5 endocytosis was markedly inhibited by 100 nM 1B7 and 500 nM RAP, but not by 100 nM 2A3 (Fig. 3A). A dose-dependent inhibition study showed a partial inhibition with 20 nM 1B7 and a similarly strong inhibition with 100 nM and 250 nM 1B7, indicating that 100 nM 1B7 is sufficient to block ADAMTS-5 endocytosis almost completely (Fig. 3B).

**Figure 4.**
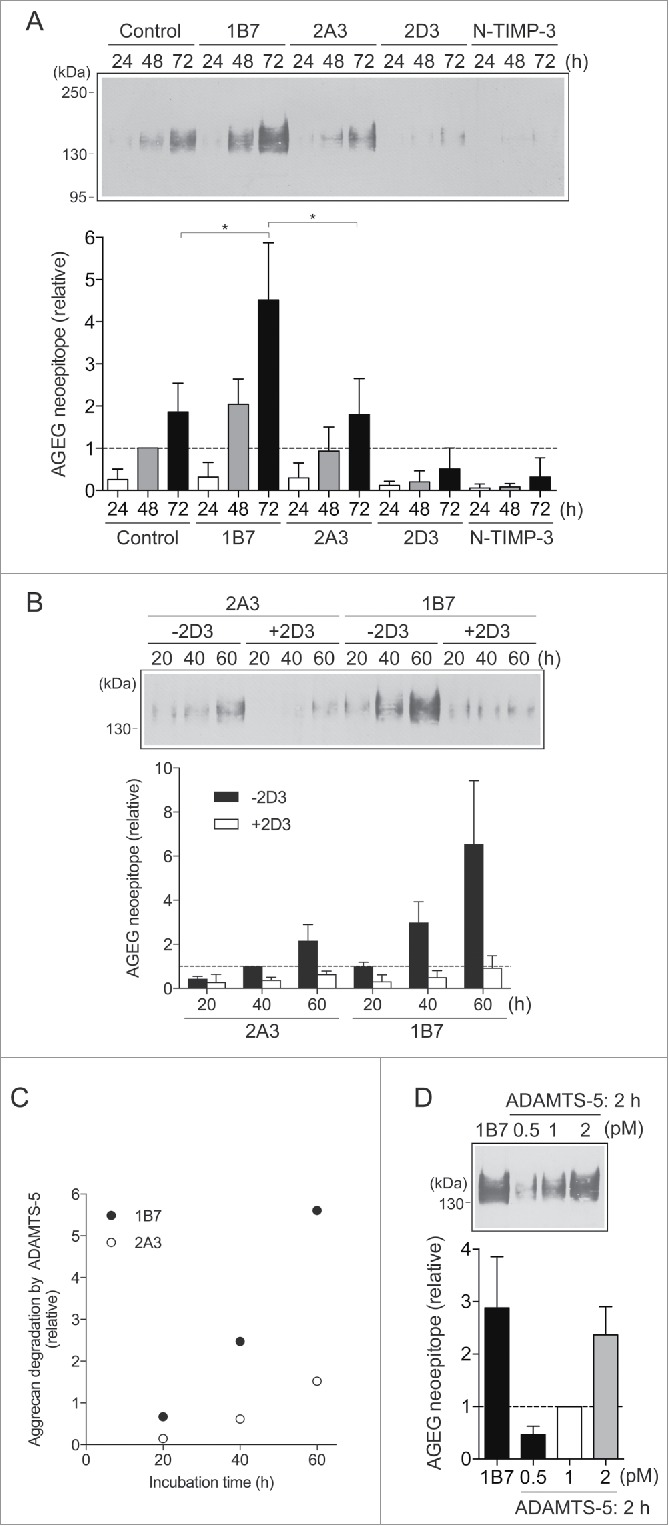
Detection of the total aggrecanolytic activity of ADAMTS-5 produced by human chondrocytes. (A) Human chondrocytes (n = 3) were cultured with DMEM containing purified bovine aggrecan (100 μg/ml) in the presence of human IgG, 1B7, 2A3, 2D3 or N-TIMP-3 (each 100 nM) for 0–72 h. The conditioned media were deglycosylated and subjected to Western blot analysis using anti-AGEG aggrecan neoepitope antibody. *Upper panel*, representative Western blot analysis. *Lower panel*, quantification of aggrecan fragments, where the mean value for the fragments generated after 48 h incubation with human IgG was taken as 1. (B) Human chondrocytes (n = 3) were cultured with DMEM containing purified bovine aggrecan (100 μg/ml) and 2A3 or 1B7 (each 100 nM) in the presence of human IgG (-2D3) or 2D3 (each 100 nM) for 0–60 h. The aggrecanolytic activity in the culture was detected as in A. *Upper panel*, representative Western blot analysis. *Lower panel*, quantification of aggrecan fragments, where the mean value for the fragments generated after 40 h incubation with 2A3 in the absence of 2D3 was taken as 1. (C) Relative amount of ADAMTS-5-mediated aggrecan degradation was estimated by subtracting the amount of aggrecan degradation in the presence of 2D3 from that in the absence of 2D3 detected in B. (D) Purified bovine aggrecan (100 μg/ml) was incubated with purified ADAMTS-5–2 (0–2 pM) for 2 h and the amount of aggecan fragments was compared with that in the medium of human chondrocytes after 72 h incubation with 1B7. The mean value for the amount of the fragments after 2 h incubation with 1 pM ADAMTS-5–2 was taken as 1. Bars represent the mean ± SD *, *p* < 0.05; unpaired *t* test.

We then investigated whether 1B7 increases the aggrecanolytic activity of the endogenously produced ADAMTS-5 by blocking its endocytosis. In these experiments, native bovine aggrecan was added to the medium of cultured human chondrocytes as substrate to detect aggrecanolytic activity, and the aggrecanase-specific cleavage of aggrecan core protein was measured by Western blotting using the anti-AGEG neoepitope antibody. Cultured chondrocytes treated with human IgG and those treated with non-inhibitory antibody 2A3 exhibited a similar time-dependent cleavage of aggrecan, which was inhibited by the N-terminal domain of tissue inhibitor of metalloproteinase-3 (N-TIMP-3) and 2D3 (Fig. 4A), an inhibitory antibody previously shown to potently inhibit aggrecanase activity.^17^ Since ADAMTS-5 is inhibited almost completely by 2D3 at the concentration of 100 nM,^17^ the residual activity detected in the presence of 2D3 was due to ADAMTSs other than ADAMTS-5. In the presence of 1B7, the aggrecan degradation was increased by about 2.4-fold after 72 h incubation compared with that with 2A3 (Fig. 4A). Quantitative mRNA analysis showed similar levels of LRP1 mRNA in the absence or presence of 1B7 (data not shown). The 1B7-increased aggrecan degradation was inhibited by 2D3 to the similar level of control chondrocytes treated with 2D3 (Fig. 4B). Subtraction of the aggrecanolytic activity of non-ADAMTS-5 aggrecanases from the total activity revealed that the increase in ADAMTS-5-mediated aggrecan degradation was much more pronounced in the presence of 1B7 compared with that in the presence of the non-inhibitory antibody 2A3 (Fig. 4C). These results indicate the extracellular level of ADAMTS-5 is constant (in a steady-state) in control chondrocytes, but the specific blocking of ADAMTS-5 endocytosis accumulates the enzyme extracellularly.

To estimate how much the ADAMTS-5 activity is regulated by endocytosis, the aggrecanolytic activity observed after 72 h incubation in the presence of 1B7 was compared with that of purified ADAMTS-5. The amount of aggrecanolytic activity accumulated in the 200-μl culture medium during 72 h from 5 × 10^4^ chondrocytes in the presence of 1B7 was similar to that detected by a 2-h incubation of 2 pM recombinant ADAMTS-5–2 (Fig. 4D).

## Discussion

Since numerous molecules are endocytosed by LRP1, an agent that selectively blocks endocytosis of a specific LRP1 ligand is considered to be a useful tool to investigate the pathophysiological roles of the molecule. In this study, we characterized a monoclonal antibody against one of the LRP1 ligands, ADAMTS-5, from a phage-displayed single-chain antibody library. The antibody 1B7 selectively blocks the interaction of the enzyme with LRP1 without affecting its aggrecanolytic activity. To date, several examples of molecules that can interfere with the binding of LRP1 ligand to the receptor have been reported. For example, Lenting *et al.*^21^ identified a monoclonal antibody that blocks the binding of coagulation factor VIII to LRP1. RNA-aptamers for pro-urokinase plasminogen activator (pro-uPA)^22^ and for tissue plasminogen activator (tPA)^23^ that inhibit their binding to LRP1 were also reported. However, the antibody inhibits the proteolytic activity of factor VIII,^21^ the former aptamers block the binding of pro-uPA to the uPA receptor and interfere with pro-uPA activation by plasmin,^22^ and the latter aptamers inhibit tPA-mediated plasminogen activation in the presence of a stimulating soluble fibrin fragment.^23^ Another set of examples is provided by segments of ligand binding domains of LRP1. The ectodomain of LRP1 harbors 4 clusters (clusters I-IV) of ligand binding sites, each composed of between 2 and 11 cysteine-rich complement-type repeats that are basic element for ligand binding. Chen *et al.*^24^ have determined that the N-terminal half of cluster II without the EGF-like domain (midkine-trap) has the highest affinity to cell growth factor midkine. This midkine-trap blocked the internalization of the growth factor and its translocation to nucleus and prevented tumor cells growth.^24^ More recently, Scilabra *et al.*^25^ reported that the same fragment also bound to TIMP-3 and prevented the LRP1-mediated internalization of TIMP-3, but not that of ADAMTS-5, ADAMTS-4 or MMP-13. At the moment, designing molecules that can selectively block ADAMTS-5 endocytosis by mimicking LRP1 binding elements remains a challenging task, since many LRP1 ligands, including ADAMTS-4 and ADAMTS-5, bind to clusters II and IV and compete with each other for the binding sites.^20^ This problem was circumvented by developing the antibody 1B7, and the treatment of human chondrocytes with 1B7 allowed us to detect full aggrecanolytic activity of ADAMTS-5 in the medium.

We originally postulated that antibodies that block the endocytosis of ADAMTS-5 would react with the first TS and Sp domains of ADAMTS-5, as these domains are responsible for LRP1 binding.^20^ However, our study using domain deletion mutants of ADAMTS-5 revealed that the 1B7 antibody binds to the Cat/Dis domains. Since it does not recognize ADAMTS-5 under reducing conditions, it is likely that it binds to a non-linear epitope. We speculate 3 possibilities to explain how 1B7 may block the interaction of ADAMTS-5 to LRP1. One possibility is that 1B7 recognizes the Cat/Dis domains of ADAMTS-5 primary, but it also interacts with the first TS, CysR and Sp domains, because the affinity of ADAMTS-5–2 is 2.1-fold higher than ADAMTS-5–5. The second possibility is that antibody 1B7 interferes with the interaction between the first TS or Sp domain and LRP1 by steric hindrance. The third possibility is that 1B7 allosterically blocks the flexibility between different ancillary domains, in a binding mode similar to that of a recently reported anti-ADAMTS-5 antibody.^5^ Future structural studies are necessary to understand these possibilities.

Selective inhibition of endocytosis and proteolytic activity of ADAMTS-5 by the combination of antibodies 1B7 and 2D3 revealed a dynamic cellular regulation of ADAMTS-5 activity, i.e., constitutive production and a rapid endocytosis. Since aggrecanolytic activity of ADAMTS-5 in the cultured human chondrocytes was exponentially increased when endocytosis was blocked, 1B7 could be useful to identify new substrates in this system and in other tissues. We propose that dysregulation of the LRP1-mediated endocytic pathway is likely to disrupt normal turnover of extracellular molecules. Indeed, we previously found that LRP1-mediated endocytosis was impaired in OA cartilage, which resulted in increased extracellular activity of ADAMTS-5 and degradation of cartilage matrix.^16^ However, the exact role of ADAMTS-5 in the development of OA is not clearly understood because the uptake of most LRP1 ligands, including ADAMTS-4, MMP-2, MMP-9, MMP-13, TIMP-3, and connective tissue growth factor (CCN-2),^15,20,26-28^ is prevented. Thus, 1B7 should be a useful tool to elucidate the pathological impact of ADAMTS-5 by selectively blocking its endocytic pathway. In contrast to OA, the overexpression of LRP1 in atherosclerotic lesions has been reported in humans and in several animal models,^29^ and the elevated ADAMTS-5 activity is considered to play an important role in turnover of vascular proteoglycans and keep the lipoprotein level low in aorta.^30^ These studies suggest a pathological role of depletion of ADAMTS-5 activity due to its excess removal from the tissue. We are currently testing whether 1B7 can penetrate the tissues because it may be beneficial in these pathological conditions, rescuing the enzyme from its endocytic clearance.

## Materials and methods

### Preparation of recombinant proteins and antibodies

Various forms of domain deleted human ADAMTS-5,^8^ human ADAMTS-4 lacking C-terminus Sp domain,^31^ TIMP-3^32^ were prepared as recombinant proteins with a FLAG-tag at C-terminus as described previously. Human N-terminal domain of TIMP-3 was prepared as reported.^32^ The concentrations of active enzymes were determined by titration against known concentrations of N-TIMP-3 using quenched-fluorescent peptides. Rabbit anti-AGEG antibodies that recognize the N-terminal AGEG generated by aggrecanase cleavage of bovine aggrecan at Glu^1771^-Ala^1772^,^33^ and RAP^16^ were prepared as reported. Bovine nasal cartilage aggrecan was prepared according to Hascall and Sajdera.^34^ The polyclonal rabbit anti-ADAMTS-5 Cat domain recognizing the peptide sequence CEETFGSTEDKRL (amino acids 410– 422) has been described previously.^8^ The anti-ADAMTS-5 antibodies were expressed in HEK293-F cells (Invitrogen) as single-chain variable fragments fused with the crystallizable fragment of immunoglobulin (scFv-Fc) and purified.^35^ Western Blue® stabilized substrate (5-bromo-4-chloro-3-indolyl-1-phosphate and nitroblue tetrazolium) for alkaline phosphatase (AP), anti-mouse AP-linked antibody (S3721), anti-human immunoglobulin AP-linked (S3821) and anti-rabbit AP-linked (S3731) antibodies were from Promega and solubilized and purified full-length human LRP1 from was from BioMac (#04–03). Unless stated otherwise, all chemicals were purchased from Sigma-Aldrich.

### Aggrecan digestion assay

Aggrecan digestion assay was performed as previously reported.^17^ Briefly, 50 μg of native aggrecan (final concentration 670 nM) were incubated with ADAMTS-5–2 (0–2 pM) in TNC buffer (50 mM Tris-HCl pH 7.5, 100 mM NaCl, 10 mM CaCl_2_ and 0.02% NaN_3_) containing 0.05% (v/v) Brij-35 at 37°C for 2 h. Aggrecan was deglycosylated in sodium acetate buffer with chondroitinase ABC and endo-β-galactosidase (each 0.01 unit/100 µg of aggrecan) for 24 h at 37°C. Aggrecan was then precipitated using ice-cold acetone and analyzed by Western blotting using the rabbit polyclonal anti-AGEG antibody. Immune signals for the aggrecan fragments were acquired using ImageScannerIII (GE Healthcare) and quantified with ImageJ quantification software.

### Phage display selections

Phage display selections using human recombinant ADAMTS-5 as an antigen were previously reported.^17^ Biotinylated ADAMTS-5–2 was used to screen a naive human scFv phage-display library.^36^ The library was previously deselected against flag peptide (5 μM) to remove phages recognizing the flag tag on the recombinant antigen. Following 2 rounds of solution-phase selection, the eluted polyclonal scFv population was cloned into pBIOCAM5 expression vector for mammalian expression of scFv–Fc fusions and transformed into DH5α *Escherichia coli*.^35^ Individual clones isolated were transfected into HEK293-F cells (Invitrogen). Conditioned medium was then screened against biotinylated recombinant ADAMTS-5–2 (50 nM). Bound antibodies were detected by time-resolved fluorescence using europium-labeled anti-human antibody (1244–330, PerkinElmer) and dissociation-enhanced lanthanide fluorescence immunoassay enhancement solution (PerkinElmer) followed by europium signal acquisition (330 nm excitation, 620 nm emission) on a plate reader. Following initial screening, selected anti-ADAMTS-5 clones were expressed in 25-ml scale and purified by immobilized metal affinity chromatography.^17^

### ELISA for binding to LRP1

Human LRP1 (5 nM in 100 µl of TNC buffer) was coated onto microtiter plates (Corning) overnight at 4 °C. Wells were blocked with 3% bovine serum albumin in TNC (1 h; 37°C) and washed in TNC containing 0.05% Brij-35 after this and each subsequent step. Wells were then incubated with various concentrations of ADAMTS-4 lacking C-terminus spacer domain, ADAMTS-5–2 or TIMP-3 in blocking solution for 2 h at room temperature. To compare the levels of bound proteins, the bound proteins were detected with anti-FLAG M2 antibody (F1804, Sigma-Aldrich) followed by anti-mouse antibody coupled to horseradish peroxidase. For screening purposes, biotinylated ADAMTS-5–2 was used and its binding to LRP1 was detected by AP-conjugated streptavidin. Hydrolysis of tetramethylbenzidine substrate (KPL) measured at 450 nm using a BioTek EL-808 absorbance microplate reader (BioTek). Data were fitted to the One-Site association equation on software package GraphPad Prism. Each value was normalized by subtracting the amount of enzyme bound to control wells that were not coated with LRP1.

### Biolayer interferometry

1B7 was biotinylated by incubation with a 20-fold excess of EZ-link succinimidyl 6-(biotinamido) hexanoate (30 min, 25°C, Thermo Fisher Scientific) and biotin excess was removed using a PD-10 desalting column (GE Healthcare). Biotinylated 1B7 (1 µM) was immobilized on streptavidin-coated biosensors (5 min, 25°C) using an Octet RED384 biolayer interferometer (Pall ForteBio). To determine the affinity between 1B7 and ADAMTS-5, antibody-coated biosensors were incubated with ADAMTS-5–2 (50–500 nM in TNC buffer) or ADAMTS-5–5 (50–1600 nM), and association was observed for 5 min at 25°C. Tips were then incubated in TNC buffer for a further 10 min at 25°C to observe dissociation of the complex. No binding of ADAMTS-5 to uncoated biosensors was observed. Kinetic constants were calculated using a 1:1 binding model in ForteBio OctetRED evaluation data analysis software as described previously.^37^ *K_D_* was calculated from equilibrium responses, and *k_off_* was calculated by determining the first-order rate constant. Observed association rate constant (*k_obs_*) values were calculated by determining the second-order rate constant, and *k_on_* was calculated from linear regression of *k_obs_* on ADAMTS-5 concentration.

### Human cartilage tissue preparation and isolation of chondrocytes

Normal human articular cartilage tissues were obtained from the Stanmore BioBank, Institute of Orthopaedics, Royal National orthopedic Hospital, Stanmore from patients following informed consent and approval by the Royal Veterinary College Ethics and Welfare Committee (Institutional approval URN 2012 0048H). Cartilage from human femoral condyles of the knee joints was used. Normal articular cartilage was obtained from patients following knee amputation due to soft tissue sarcoma and osteosarcoma with no involvement of the cartilage. Tissues were obtained from 5 patients (3 males aged 18, 23 and 57 yrs; 2 females aged 19 and 68 yrs). Chondrocytes were isolated as described previously.^16^ Both primary and passaged human cells were used in the experiments.

### Analysis of ADAMTS-5 endocytosis

Cells (5 × 10^4^) cultured in 24-well plates were rested in 500 μl of Dulbecco's Modified Eagle Medium (DMEM) for 1 day. The medium was replaced with 500 μl of fresh DMEM with 10 nM of purified human ADAMTS-5–2 in the absence or presence of the antibodies (each 100 nM) or 500 nM RAP at 37°C. After incubation for 0–4 h, media were collected and the protein was precipitated with 5% trichloroacetic acid and dissolved in 50 μl of 1x SDS-sample buffer containing 5% 2-mercaptoethanol. All samples were analyzed by SDS-PAGE under reducing conditions and Western blotting using anti-FLAG M2 mouse monoclonal antibody. Immune signals for exogenously added ADAMTS-5–2 detected in the medium were acquired using ImageScannerIII (GE Healthcare) and quantified with ImageJ quantification software. The amount of ADAMTS-5–2 remaining in the medium at each time point was calculated as a percentage of the amount of the enzyme at 0 h.

### Detection of aggrecanolytic activity in cultured human chondrocytes

The aggrecanase activity in culture medium of human chondrocytes was detected as described previously.^38^ Briefly, cells were plated at a density of 5×10^4^ cells/well (24-well plate) in DMEM with 10% fetal bovine serum. Cells were rested for 24 h in DMEM and then overlaid with sterile filtered bovine aggrecan (100 μg/ml) in DMEM containing 50 µg/ml of polymyxinB in the absence or presence of the antibodies or N-TIMP-3 (each 100 nM) for 0–72 h. To evaluate aggrecan degradation products in chondrocytes cultures, 200 μl of conditioned medium were collected and aggrecanase-generated fragments were detected by Western blot analysis as described above. All data were analyzed by unpaired one-tail t tests with Welch's correction using the software package GraphPad Prism.

## Fundings

The work was supported by Arthritis Research UK (20563), Arthritis Research UK Center for Osteoarthritis Pathogenesis (20205), Cancer Research UK (C100/A8243), the Kennedy Trust for Rheumatology Research, orthopedic Research UK (483) and the National Institute of Arthritis and Musculoskeletal and Skin Diseases (AR40994).

## References

[1] Fushimi K, Troeberg L, Nakamura H, Lim NH, Nagase H Functional Differences of the Catalytic and Non-catalytic Domains in Human ADAMTS-4 and ADAMTS-5 in Aggrecanolytic Activity. J Biol Chem 2008; 283:6706-16; PMID:18156631; http://dx.doi.org/10.1074/jbc.M708647200 18156631

[2] Durigova M, Nagase H, Mort JS, Roughley PJ MMPs are less efficient than ADAMTS5 in cleaving aggrecan core protein. Matrix Biol 2011; 30:145-53; PMID:21055468; http://dx.doi.org/10.1016/j.matbio.2010.10.007 21055468 PMC3057330

[3] Glasson SS, Askew R, Sheppard B, Carito B, Blanchet T, Ma HL, Flannery CR, Peluso D, Kanki K, Yang Z, et al. Deletion of active ADAMTS5 prevents cartilage degradation in a murine model of osteoarthritis. Nature 2005; 434:644-8; PMID:15800624; http://dx.doi.org/10.1038/nature03369 15800624

[4] Stanton H, Rogerson FM, East CJ, Golub SB, Lawlor KE, Meeker CT, Little CB, Last K, Farmer PJ, Campbell IK, et al. ADAMTS5 is the major aggrecanase in mouse cartilage in vivo and in vitro. Nature 2005; 434:648-52; PMID:15800625; http://dx.doi.org/10.1038/nature03417 15800625

[5] Larkin J, Lohr TA, Elefante L, Shearin J, Matico R, Su JL, Xue Y, Liu F, Genell C, Miller RE, et al. Translational development of an ADAMTS-5 antibody for osteoarthritis disease modification. Osteoarthritis Cartilage 2015; 23:1254-66; PMID:25800415; http://dx.doi.org/10.1016/j.joca.2015.02.778 25800415 PMC4516626

[6] McCulloch DR, Le Goff C, Bhatt S, Dixon LJ, Sandy JD, Apte SS Adamts5, the gene encoding a proteoglycan-degrading metalloprotease, is expressed by specific cell lineages during mouse embryonic development and in adult tissues. Gene Expr Patterns 2009; 9:314-23; PMID:19250981; http://dx.doi.org/10.1016/j.gep.2009.02.006 19250981 PMC2725439

[7] Stupka N, Kintakas C, White JD, Fraser FW, Hanciu M, Aramaki-Hattori N, Martin S, Coles C, Collier F, Ward AC, et al. Versican Processing by a Disintegrin-like and Metalloproteinase Domain with Thrombospondin-1 Repeats Proteinases-5 and -15 Facilitates Myoblast Fusion. J Biol Chem 2013; 288:1907-17; PMID:23233679; http://dx.doi.org/10.1074/jbc.M112.429647 23233679 PMC3548499

[8] Gendron C, Kashiwagi M, Lim NH, Enghild JJ, Thogersen IB, Hughes C, Caterson B, Nagase H Proteolytic Activities of Human ADAMTS-5: COMPARATIVE STUDIES WITH ADAMTS-4. J Biol Chem 2007; 282:18294-306; PMID:17430884; http://dx.doi.org/10.1074/jbc.M701523200 17430884

[9] Dupuis LE, McCulloch DR, McGarity JD, Bahan A, Wessels A, Weber D, Diminich AM, Nelson CM, Apte SS, Kern CB Altered versican cleavage in ADAMTS5 deficient mice; a novel etiology of myxomatous valve disease. Dev Biol 2011; 357:152-64; PMID:21749862; http://dx.doi.org/10.1016/j.ydbio.2011.06.041 21749862 PMC4435578

[10] Longpre JM, McCulloch DR, Koo BH, Alexander JP, Apte SS, Leduc R Characterization of proADAMTS5 processing by proprotein convertases. Int J Biochem Cell Biol 2009; 41:1116-26; PMID:18992360; http://dx.doi.org/10.1016/j.biocel.2008.10.008 18992360

[11] McCulloch DR, Nelson CM, Dixon LJ, Silver DL, Wylie JD, Lindner V, Sasaki T, Cooley MA, Argraves WS, Apte SS ADAMTS metalloproteases generate active versican fragments that regulate interdigital web regression. Dev Cell 2009; 17:687-98; PMID:19922873; http://dx.doi.org/10.1016/j.devcel.2009.09.008 19922873 PMC2780442

[12] Hattori N, Carrino DA, Lauer ME, Vasanji A, Wylie JD, Nelson CM, Apte SS ericellular versican regulates the fibroblast-myofibroblast transition: a role for ADAMTS5 protease-mediated proteolysis. J Biol Chem 2011; 286:34298-310; PMID:21828051; http://dx.doi.org/10.1074/jbc.M111.254938 21828051 PMC3190794

[13] McMahon M, Ye S, Izzard L, Dlugolenski D, Tripp RA, Bean AG, McCulloch DR, Stambas J ADAMTS5 Is a Critical Regulator of Virus-Specific T Cell Immunity. PLoS Biol 2016; 14:e1002580; PMID:27855162; http://dx.doi.org/10.1371/journal.pbio.1002580 27855162 PMC5113859

[14] Troeberg L, Nagase H Proteases involved in cartilage matrix degradation in osteoarthritis. Biochim Biophys Acta 2012; 1824:133-145; PMID:21777704; http://dx.doi.org/10.1016/j.bbapap.2011.06.020 21777704 PMC3219800

[15] Yamamoto K, Murphy G, Troeberg L Extracellular regulation of metalloproteinases. Matrix Biol 2015; 44-46:255-63; PMID:25701651; http://dx.doi.org/10.1016/j.matbio.2015.02.007 25701651

[16] Yamamoto K, Troeberg L, Scilabra SD, Pelosi M, Murphy CL, Strickland DK, Nagase H LRP-1-mediated endocytosis regulates extracellular activity of ADAMTS-5 in articular cartilage. FASEB J 2013; 27:511-21; PMID:23064555; http://dx.doi.org/10.1096/fj.12-216671 23064555 PMC3545526

[17] Santamaria S, Yamamoto K, Botkjaer K, Tape C, Dyson MR, McCafferty J, Murphy G, Nagase H Antibody-based exosite inhibitors of ADAMTS-5 (aggrecanase-2). Biochem J 2015; 471:391-401; PMID:26303525; http://dx.doi.org/10.1042/BJ20150758 26303525 PMC4613496

[18] Lillis AP, Van Duyn LB, Murphy-Ullrich JE, Strickland DK LDL receptor-related protein 1: unique tissue-specific functions revealed by selective gene knockout studies. Physiol Rev 2008; 88:887-918; PMID:18626063; http://dx.doi.org/10.1152/physrev.00033.2007 18626063 PMC2744109

[19] Koza RA, Nikonova L, Hogan J, Rim JS, Mendoza T, Faulk C, Skaf J, Kozak LP Changes in gene expression foreshadow diet-induced obesity in genetically identical mice. PLoS Genet 2006; 2:e81; PMID:16733553; http://dx.doi.org/10.1371/journal.pgen.0020081 16733553 PMC1464831

[20] Yamamoto K, Owen K, Parker AE, Scilabra SD, Dudhia J, Strickland DK, Troeberg L, Nagase H Low density lipoprotein receptor-related protein 1 (LRP1)-mediated endocytic clearance of a disintegrin and metalloproteinase with thrombospondin motifs-4 (ADAMTS-4): functional differences of non-catalytic domains of ADAMTS-4 and ADAMTS-5 in LRP1 binding. J Biol Chem 2014; 289:6462-74; PMID:24474687; http://dx.doi.org/10.1074/jbc.M113.545376 24474687 PMC3945312

[21] Lenting PJ, Neels JG, van den Berg BM, Clijsters PP, Meijerman DW, Pannekoek H, van Mourik JA, Mertens K, van Zonneveld AJ The light chain of factor VIII comprises a binding site for low density lipoprotein receptor-related protein. J Biol Chem 1999; 274:23734-9; PMID:10446132; http://dx.doi.org/10.1074/jbc.274.34.23734 10446132

[22] Dupont DM, Thuesen CK, Botkjaer KA, Behrens MA, Dam K, Sorensen HP, Pedersen JS, Ploug M, Jensen JK, Andreasen PA Protein-binding RNA aptamers affect molecular interactions distantly from their binding sites. PLoS ONE 2015; 10:e0119207; PMID:25793507; http://dx.doi.org/10.1371/journal.pone.0119207 25793507 PMC4368798

[23] Bjerregaard N, Botkjaer KA, Helsen N, Andreasen PA, Dupont DM Tissue-type plasminogen activator-binding RNA aptamers inhibiting low-density lipoprotein receptor family-mediated internalisation. Thromb Haemost 2015; 114:139-49; PMID:25855589; http://dx.doi.org/10.1160/TH14-08-0686 25855589

[24] Chen S, Bu G, Takei Y, Sakamoto K, Ikematsu S, Muramatsu T, Kadomatsu K Midkine and LDL-receptor-related protein 1 contribute to the anchorage-independent cell growth of cancer cells. J Cell Sci 2007; 120:4009-015; PMID:17971413; http://dx.doi.org/10.1242/jcs.013946 17971413

[25] Scilabra SD, Yamamoto K, Pigoni M, Sakamoto K, Muller SA, Papadopoulou A, Lichtenthaler SF, Troeberg L, Nagase H, Kadomatsu K Dissecting the interaction between tissue inhibitor of metalloproteinases-3 (TIMP-3) and low density lipoprotein receptor-related protein-1 (LRP-1): Development of a “TRAP” to increase levels of TIMP-3 in the tissue. Matrix Biol 2016; 59:69-79; PMID:27476612; http://dx.doi.org/23166318 10.1016/j.matbio.2016.07.004 27476612

[26] Scilabra SD, Troeberg L, Yamamoto K, Emonard H, Thogersen I, Enghild JJ, Strickland DK, Nagase H Differential regulation of extracellular tissue inhibitor of metalloproteinases-3 levels by cell membrane-bound and shed low density lipoprotein receptor-related protein 1. J Biol Chem 2013; 288:332-342; PMID:23166318; http://dx.doi.org/10.1074/jbc.M112.393322 23166318 PMC3537031

[27] Yamamoto K, Okano H, Miyagawa W, Visse R, Shitomi Y, Santamaria S, Dudhia J, Troeberg L, Strickland DK, Hirohata S, et al. MMP-13 is constitutively produced in human chondrocytes and co-endocytosed with ADAMTS-5 and TIMP-3 by the endocytic receptor LRP1. Matrix Biol 2016; 56:57-73; PMID:27084377; http://dx.doi.org/10.1016/j.matbio.2016.03.007 27084377 PMC5146981

[28] Kawata K, Kubota S, Eguchi T, Aoyama E, Moritani NH, Kondo S, Nishida T, Takigawa M Role of LRP1 in transport of CCN2 protein in chondrocytes. J Cell Sci 2012; 125:2965-72; PMID:22454511; http://dx.doi.org/10.1242/jcs.101956 22454511

[29] Llorente-Cortes V, Badimon L LDL receptor-related protein and the vascular wall: implications for atherothrombosis. Arterioscler Thromb Vasc Biol 2005; 25:497-04; PMID:15705932; http://dx.doi.org/10.1161/01.ATV.0000154280.62072.fd 15705932

[30] Didangelos A, Mayr U, Monaco C, Mayr M Novel role of ADAMTS-5 protein in proteoglycan turnover and lipoprotein retention in atherosclerosis. J Biol Chem 2012; 287:19341-45; PMID:22493487; http://dx.doi.org/10.1074/jbc.C112.350785 22493487 PMC3365970

[31] Kashiwagi M, Enghild JJ, Gendron C, Hughes C, Caterson B, Itoh Y, Nagase H Altered Proteolytic Activities of ADAMTS-4 Expressed by C-terminal Processing. J Biol Chem 2004; 279:10109-19; PMID:14662755; http://dx.doi.org/10.1074/jbc.M312123200 14662755

[32] Troeberg L, Fushimi K, Scilabra SD, Nakamura H, Dive V, Thogersen IB, Enghild JJ, Nagase H The C-terminal domains of ADAMTS-4 and ADAMTS-5 promote association with N-TIMP-3. Matrix Biol 2009; 28:463-69; PMID:19643179; http://dx.doi.org/10.1016/j.matbio.2009.07.005 19643179 PMC2835468

[33] Troeberg L, Fushimi K, Khokha R, Emonard H, Ghosh P, Nagase H Calcium pentosan polysulfate is a multifaceted exosite inhibitor of aggrecanases. FASEB J 2008; 22:3515-24; PMID:18632849; http://dx.doi.org/10.1096/fj.08-112680 18632849 PMC2537431

[34] Hascall VC, Sajdera SW Proteinpolysaccharide complex from bovine nasal cartilage. The function of glycoprotein in the formation of aggregates. J Biol Chem 1969; 244:2384-96; PMID:578384022842086 5783840

[35] Falk R, Falk A, Dyson MR, Melidoni AN, Parthiban K, Young JL, Roake W, McCafferty J Generation of anti-Notch antibodies and their application in blocking Notch signalling in neural stem cells. Methods 2012; 58:69-78; PMID:22842086; http://dx.doi.org/10.1016/j.ymeth.2012.07.008 22842086 PMC3502869

[36] Schofield DJ, Pope AR, Clementel V, Buckell J, Chapple S, Clarke KF, Conquer JS, Crofts AM, Crowther SR, Dyson MR, et al. Application of phage display to high throughput antibody generation and characterization. Genome Biol 2007; 8:R254; PMID:18047641; http://dx.doi.org/25176127 10.1186/gb.2007.8.11.r254 18047641 PMC2258204

[37] Troeberg L, Lazenbatt C, Anower-E-Khuda MF, Freeman C, Federov O, Habuchi H, Habuchi O, Kimata K, Nagase H Sulfated glycosaminoglycans control the extracellular trafficking and the activity of the metalloprotease inhibitor TIMP-3. Chem Biol 2014; 21(10): 1300-09; PMID:25176127; http://dx.doi.org/10.1016/j.chembiol.2014.07.014 25176127 PMC4210636

[38] Ismail HM, Yamamoto K, Vincent TL, Nagase H, Troeberg L, Saklatvala J Interleukin 1 acts via c-jun N-terminal kinase-2 signalling pathway to induce aggrecan degradation by human chondrocytes. Arthritis Rheumatol 2015; 67(7): 1826-36; PMID:25776267; http://dx.doi.org/10.1002/art.39099 25776267

